# Help-seeking strategies and treatment experiences among individuals diagnosed with Bipolar Spectrum Disorder in Iran: A qualitative study

**DOI:** 10.1177/13634615221127855

**Published:** 2022-10-17

**Authors:** Fahimeh Mianji, Laurence J. Kirmayer

**Affiliations:** 1 Division of Social and Transcultural Psychiatry, Department of Psychiatry, 5620 McGill University, Canada; 2 Culture and Mental Health Research Unit, Institute of Community and Family Psychiatry, Canada

**Keywords:** bipolar spectrum disorder, help-seeking, Iran, mental healthcare system, structural and cultural competence

## Abstract

Social, cultural, and structural factors are associated with delays in seeking help from mental health professionals and poor treatment adherence among patients with mood disorders. This qualitative study examined the perspectives on the services and response to treatments of individuals diagnosed with Bipolar Spectrum Disorder (BSD) in Iran through 37 in-depth semi-structured interviews with patients who had received BSD diagnosis and treatment (excluding Bipolar-I). Interviews explored two broad areas: 1) coping and help-seeking strategies; and 2) barriers to treatment and expectations of outcomes from treatment. Multiple factors influenced the help-seeking strategies and trajectories of patients with BSD diagnoses in Iran, including: structural limitations of the mental healthcare system; modes of practice of biological psychiatry; characteristics of the official psychology and counseling services permitted by Iran’s government; popular psychology and consultation (offered through social media from the diaspora) by Iranian psychologists and counsellors in the diaspora; and alternative spiritual and cult-based groups. To improve the quality and accessibility of mental health services, it is essential to have structural changes in the healthcare system that prioritize human rights and individuals’ values over the political and ideological values of the state and changes in the professions that promote secular training of mental healthcare providers and an ecosocial model of care.

## Introduction

Social and cultural variations in the experience of mental health problems, coping strategies, help-seeking behaviors, clinical presentations, and responses to interventions influence the treatment and recovery of patients with mental disorders (Kirmayer et al., 2013). Several studies in diverse settings have documented delays in seeking help from mental health professionals and poor treatment adherence among patients with mood disorders (Brandstetter et al., 2017; Eftekhari et al., 2014; Mojtabai et al., 2011). Social, cultural, and structural factors are associated with problems accessing appropriate interventions for patients with mental health issues. These factors include stigma, self-reliance and low perceived need for care, differences between lay explanations of mental health problems and explanations offered by biomedicine, negative attitudes towards psychiatric medications and hospitalization, confidentiality issues, lack of proper investigation and nonspecific care, lack of clinicians’ cultural competence, minimal knowledge about mental health problems and existing mental health services, and costs and availability of mental health services (Clement et al., 2015; Dejman et al., 2009; Dockery et al., 2015; Eftekhari et al., 2014; Evans-Lacko et al. 2012; Mojtabai et al. 2011; Taghva et al., 2017).

For serious mental health problems, people in non-Western countries often pursue both biomedical and ethnomedical healing modalities (Chang et al., 2014; Hsu et al., 2009; Kleinman, 1980; Luu et al., 2009; Shih et al., 2010). For example, seeking help from traditional Chinese medicine practitioners and engagement with religious and spiritual practices for physical or emotional concerns is commonly reported among patients with bipolar disorder in Taiwan (Lan et al., 2018). Similarly, studies on pathways to care among patients with mental disorders in Iran show that the majority of patients seek help from both medical and traditional healers. In a study of explanatory models of depression and pathways to care among female patients from three ethnic groups (Fars, Kurds, and Turks) in Iran, the majority of patients reported visiting several non-psychiatric physicians (such as general practitioners and other specialists) before they were referred to psychiatrists (Dejman et al., 2011). The reasons reported by patients for not visiting psychiatrists and psychologists included concern about stigmatization and the affordability of psychological services. Among those who did consult psychiatrists, some patients did not continue in treatment because of drug side effects, concerns that medication had aggravated their illness, the long duration of treatment, and the time it took for medication to be effective. Other help-seeking strategies, such as spiritual and religious support, consulting fortune-tellers and prayer-writers, and collective practices such as Zar in the south of Iran, were reported to be common among patients with mental health disorders (Dejman et al., 2009; Mianji & Semnani, 2015).

Across diverse contexts, patients with bipolar disorders commonly express concerns about stigma, over-medication, side effects, issues regarding the patient–clinician relationship, and the affordability and accessibility of services (Chakrabarti, 2016; Jawad et al., 2018; Lingam & Scott, 2002; Perlick et al., 2004; Thompson & McCabe 2012; Zeber et al., 2008). However, there are specific cultural, social, and political determinants that shape patients’ experience and understanding of their symptoms and suffering, causal attributions, strategies for seeking help, and expectations about care. For instance, spiritual and sociomoral understandings of mental illness and parallel help-seeking strategies are frequently employed in Asian countries (e.g., Chi et al., 1996; Kleinman, 1980). However, these local cultural models and approaches are not uniformly endorsed within particular social and cultural settings. For example, a recent study on explanatory models for bipolar disorder in Taiwan found generational disagreements in families regarding adherence to Daoist and Christian practice, traditional Chinese medicine, and modern psychiatry (Lan et al., 2018). Similarly, generational differences in understanding, coping with, and seeking help for mental health problems are reported in studies in Iran. This is associated with a move from traditional pathways towards more biomedical interventions and popular psychology methods (Behrouzan, 2016; Fatemi et al., 2015; Mianji & Kirmayer, 2021).

Despite the rise in the use of bipolar spectrum disorder (BSD) diagnoses and corresponding biomedical interventions in Iran, associated with the globalization of American psychiatric nosology in the past two decades (Mianji & Kirmayer, 2020), there has been no study of patients’ experiences with the interventions. This paper examines help-seeking strategies and treatment experiences among individuals diagnosed with BSD in Iran. In conjunction with our previous studies of professional diagnostic practices and case formulation (Mianji & Kirmayer, 2021), the overall aim is to better understand the impact of a globalized psychiatry on mental health practice in Iran and the dilemmas created by a clinical psychiatric approach that does not integrate structural factors related to local social-cultural contexts.

## Method

This paper is a part of a larger study of the rise of BSD diagnoses in Iran that included three periods of fieldwork from February 2014 to June 2015 in four cities: Tehran, Isfahan, Shiraz, and Zabol. The study protocol was approved by the Research Ethics Committee (REC) of the Jewish General Hospital in Montreal. Earlier components examined institutional history, and practitioner perspectives. The present study focuses on patients’ experiences of diagnosis and treatment.

We used purposive sampling to identify patients with a diagnosis of BSD, who were currently attending psychiatric practices (male or female, 18–55 years old) in Tehran, Isfahan, Shiraz, and Zabol. Tehran, Isfahan, and Shiraz are among the five largest cities in Iran; Zabol is a smaller city that lies on the border with Afghanistan. Due to disparities in the distribution of the health and social resources in Iran (Chavehpour et al., 2019; Reshadat et al., 2019), there are greater barriers to accessing healthcare resources in smaller cities, rural areas, and cities along the nation’s borders compared to cities with larger populations and those located in the center of the country. By collecting data from cities with different possibilities and limitations in access to healthcare and social resources, we aimed to capture the diversity in patients’ experiences with their mental health problems and help-seeking strategies. Additionally, all of the above mentioned cities, except Zabol, have medical universities with psychiatric training programs. This facilitated professional networking and collaboration with academic psychiatrists who agreed to refer their patients for the research interview.

The study sample included 37 patients who had received BSD diagnosis and treatment, excluding Bipolar-I, both with and without a past history of hospitalization. All participants had either directly reached out or were referred to psychiatrists to treat their mental health problems. Patients had received a BSD diagnosis and treatment (excluding Bipolar-I) and were recruited by psychiatrists specialized in treating mood disorders, including psychiatrists who participated in the larger study.

Data collection was based on qualitative interviews using the McGill Illness Narrative Interview (MINI) (Groleau et al., 2006). The MINI is a semi-structured interview protocol with three main sections: 1) a basic temporal narrative of symptom and illness experience, organized in terms of the contiguity of events; 2) salient prototypes related to current health problems, based on previous experience of the interviewee, family members or friends, and mass media or other popular illness representations; and 3) explanatory models, including labels, causal attributions, expectations for treatment, and course and outcome. Supplementary sections of the MINI explore help-seeking and pathways to care, treatment experience, adherence and impact of the illness on identity, self-perception, and relationships with others (Groleau et al., 2006). In a previous paper (Mianji & Kirmayer, 2021), we presented findings from sections of the MINI, including the patients’ illness experiences and explanatory models. In this paper, we focus on the findings based on data from the supplementary sections of the MINI that addressed help-seeking history and experience. To adapt the MINI to the Iranian setting, the English version was translated into Farsi by a bilingual Iranian psychologist and was edited by a bilingual Iranian psychiatrist with experience working with Farsi-speaking patients both within and outside of the country. The interview guideline did not require modifications because of the similarity in the clinical language used by psychiatrists in Iran. This reflects the influence of American psychiatry in psychiatric training and practices in Iran (Mianji & Kirmayer, 2020).

Potential participants were identified by their psychiatrists, who participated in the first phase of the study on institutional history, as well as other psychiatrists who were former colleagues of the first author (a clinical psychologist who trained and worked in Iran and Canada). The aim of this research project was introduced to participants first by their clinicians as a study to better understand patients’ experience with their mental health problems (emphasizing mood disorders). Patients who agreed to participate were interviewed by the first author at their psychiatrists’ office, their home, or their workplace, as they preferred. All patients gave informed consent, either written or verbally. To protect participant anonymity, pseudonyms have been used in the presentation of findings.

All interviews and conversations were conducted in Farsi and ranged from one to three hours in duration. With the permission of the participants, the interviews were audio-recorded and transcribed. Transcripts were analyzed by thematic coding with an emphasis on identifying the underlying ideas, assumptions, metaphors or analogies, conceptualizations, and help-seeking strategies, based on three modes of reasoning about or representation of symptoms and help-seeking choices explored in the MINI, including exploratory models, prototypes, and chain-complexes (Groleau et al., 2006; Stern & Kirmayer, 2004).

Data quality, validity, and rigor were ensured by multiple strategies (Morse, 2015): collecting data from cities with different demographics and different levels of access to the healthcare and social resources; using multiple sources of data, including semi-structured interviews with psychiatrists (during the first phase of the project) and patients (during the second phase of the project), the analysis of archival materials and data derived from field notes during participant observation in Iranian psychiatric settings and conferences; and the content analysis of national and social media about the current mental health conditions of Iranians, which provided information on relevant help-seeking pathways. Other ethnographic data collected during field work and through social media platforms on concurrent professional and public debates were used to clarify and interpret material derived from the interviews based on the local contexts in which the narratives were produced.

## Results

In an earlier article (Mianji & Kirmayer, 2021), we elaborated on patients’ experiences with and causal attributions of their mental health problems. Most participants explained their mental health problem as a form of neurologic illness (*narahati-asāb*), depression (*afsordegi*), or “pendular” depression (*afsordegi navasāni*; i.e., depression with mood swings). However, the most common term they used for their mental health conditions was *dō-shakhsiaty boodan*, which literally means “having two personalities.” We found wide variation in patients’ understanding of their BSD diagnosis. Only a few participants, mostly those with higher education and living in Tehran, named their condition “bipolar (spectrum) disorder”; participants from more traditional backgrounds and those with lower education levels mostly framed their problem as some form of depression. Using other terms to explain their mental health problems, particularly calling it “*afsordegi*” (depression) or “*afsordegi navasāni*” (pendular depression), seemed to be associated with avoiding the stigma attached to severe psychiatric diagnoses, including bipolar disorders. There was no gender difference in the diagnostic labels or expressions used by participants with BSD. Also, the majority of participants, both male and female, attributed the causes of their mental health problems to external stressors (Mianji & Kirmayer, 2021). This paper focuses on patients’ perspectives of the services and response to treatments. In this regard, two broad areas were identified: 1) coping and help-seeking strategies; 2) barriers to treatment and expectations of outcomes from treatment.

## Coping and help-seeking strategies

Coping and help-seeking strategies reported by the study participants usually included psychiatric services and, in a few cases, psychological services, social support, and alternative sources of care. We found a discrepancy between patients’ commonly expressed causal attributions and their main help-seeking strategies. Although patients attributed their mental health problems mostly to external psychosocial factors such as familial and sociocultural stress, they sought help mainly from biomedical practitioners, including psychiatrists. Participants attributed this discrepancy to the lack of affordable, professional psychological services, the lack of safe and non-intrusive social support systems, and the social stigma of mental health problems. Although all participants sought help from the medical system, patients in different cities and patients from different educational and social backgrounds reported different strategies in coping with their mental health problems, reflecting different local demography, culture, and social context. The sections below illustrate the different help-seeking strategies reported by the participants in four cities in Iran: Tehran, Isfahan, Shiraz, and Zabol.

## Medical and psychological services

Consulting medical doctors—which include general physicians (GPs), neurologists, and psychiatrists—was identified as the most common, but not the initial, step in the help-seeking pathway of participants in the large cities—Tehran, Isfahan, and Shiraz—and one of the common pathways among participants in Zabol. Due to the stigma of mental illnesses and negative lay attitudes towards using psychiatric medications, participants expressed a preference to see a general practitioner or neurologist (*motekhases-e-maghz va asâb*) before considering visiting a psychiatrist (*ravân-pezeshk*) or psychologist (*ravân-shenas*). The lack of a referral system in Iran’s healthcare allows patients to choose which specialist to see when they need medical services. This facilitates seeing a neurologist instead of a psychiatrist for psychological problems (Mianji & Kirmayer, 2020). Compared with *maghz* (brain) and *asâb* (nerves), in Farsi, the term *ravân* (soul, psyche) is associated with the term *ravâni*, which is used interchangeably with *divâneh*, which means “mad” or “crazy.” Therefore, seeing *ravân-pezeshk* could be perceived as being *ravâni*, and that adds to the stigma of mental illness. Also, *mošâver (*counselor) is a more common term to refer to *ravân-shenas* (psychologists), the latter of which carries a stigma similar to that attached to the term *ravân* (soul, psyche) like *ravân-pezeshk* (psychiatrist). The following excerpts from interviews illustrate some of the dilemmas influencing the initial steps of seeking help within the healthcare system:

I was agitated, nervous, and aggressive. I went to see a neurologist. I told the doctor that my head is busy [thinks too much] and I have *hojoum-e afkâr* [intrusive thoughts]. I told him that it does not make sense to have such a busy head. The doctor got an EEG [electroencephalogram] and diagnosed that I have migraines, too; he prescribed me a few medications including Depakote [divalproex sodium^1^]. I used them for three months and felt slightly better, but I stopped them and decided to help myself with heavy exercise, which helped, and I became fine after. A few years later, again, I became sensitive to words, felt disappointment, and had thoughts of failing [negative thoughts]. This time, when I was visiting a book exhibition, accidentally, I saw a handbook of depression which recommended seeing a psychiatrist in case of having the symptoms that I was suffering from. I decided to see a psychiatrist. Despite knowing that in Iran when you talk about psychiatrists and psychologists everyone thinks you are crazy, I decided to follow that path and see a psychiatrist. I was prescribed different medications that made me like a dead body and I could not move. I meant to continue the meds, but, at some point, I decided to help myself with exercise instead of meds, which again helped. (Participant A, 34-year-old male, technician)

Here, the patient’s experience of his distress was reduced to biomedical causes and interventions, which did not seem to correspond to his psychological needs and consequently led him to stop the treatment. Describing symptoms of worrying in the context of “thinking too much” is a common idiom of distress used by local populations in different parts of the world (Kaiser et al., 2015). Exploring the individual and cultural meaning and use of local idioms of distress, such as “thinking a lot” or “thinking too much,” is essential to determine how suffering is understood and treated (Good et al., 1985; Hinton and Good, 2016). The lack of an integrative, multilevel model that takes into account the local and personal concepts of mental health as well the impact of the biological, psychological, and social processes may result in dismissing the patient’s perspective on the causes of distress, which can undermine the clinical alliance and treatment adherence.

Many participants reported that they saw a neurologist before visiting a psychiatrist or psychologist.

I went to see a neurologist because there is no control on [the quality of services of] counselors. I preferred to see a neurologist to get medications if needed and, if not, to get the counseling from him [the neurologist^2^]. (Participant B, 34-year-old female, business consultant)

Although patients preferred to consult GPs and neurologists initially, most ended up seeing a psychiatrist, both because they did not respond well to the medications prescribed by GPs and neurologists and because they hoped that a psychiatrist might listen to their experience of mental health problems before prescribing medications. Unfortunately, according to the majority of our participants, their need for a safe space to share their mental health problems and concerns was not met by psychiatrists either. Participants reported that their psychiatric visits were too brief—typically 5–10 min in duration—and were focused only on inquiry about some of their symptoms, which left them feeling unheard, but carrying away prescriptions for multiple medications: “In visiting my psychiatrist, I got to explain my symptoms briefly, then I was prescribed imipramine, amitriptyline, etc., [multiple tricyclic antidepressant medications], and the doctor kept changing the dosages of my medications” (Participant C, 39-year-old, female, physician). In general, the most common mode of psychiatric practice was pharmacological, focusing on symptoms and medications, with little or no attention to patients’ understanding of the causes or consequences of their suffering, leaving them feeling unattended and misunderstood.

Despite frequently expressing the need and wish to talk to a professional about their mental health experiences to be understood and receive help, the majority of our participants expressed dissatisfaction with and little trust in the psychological services, including those provided by psychologists, psychotherapists, and counselors. Their dissatisfaction was mainly attributed to the high costs and poor quality of these services. The poor quality was associated with the problems with professional and scientific accountability of the services and with the concern that clinicians projected their own values and ideological beliefs onto patients.

I tried counseling a few times, but what I saw was more like a business than therapy. One session they would ask me to do tests, one session to come back and get the test results. … [A]fter a few sessions back and forth, they told me that I have severe anxiety or anger issues. I told them that I know I have these problems, and that’s why I am seeking therapy. (Participant D, 27-year-old female, MA student)

Participants emphasized that the cost and inefficiency of private services, including the business-oriented aspect of the services, were among the main reasons for not pursuing psychological services. Some participants also attributed their lack of trust in the psychotherapy and counseling services to having received advice that reflected the therapists’ traditional and religious beliefs instead of taking the patients’ own cultural beliefs into account. Some called this way of practice “auntie-therapy,” referring to traditional ways that older family members would advise them about their life struggles.

Once, I accompanied my friend to see a psychotherapist. I think she was a physician who got a psychotherapy permit. My friend is not religious at all, and the therapist was religious. It was as if she was advising a religious person when she talked to my friend. She [the therapist] said, “I prayed to Imam Hossein^3^ to get me a husband and it worked.” It is ridiculous even to someone like me, who is not as non-religious as my friend, to ask Imam Hossein to give you a husband! (Participant B, 34-year-old, female, business consultant)

The examples given by participants in our study illustrate some of the cultural and generational tensions between young people and mental healthcare providers, such as counselors, who impose their personal views and the government’s agenda in terms of social norms about romantic and sexual behaviors on their patients instead of helping patients better navigate tensions, contradictions, and rigidity in their social environment throughout the process of resolving their mental health problems.

In summary, in terms of psychological interventions, the majority of participants expressed dissatisfaction towards both psychiatrists (for not listening and just prescribing medications) and psychologists and counselors (for imposing another normative culture, consistent with their religious and ideological views). In both ways, psychological care was seen as a form of silencing the individual’s own cultural and social beliefs and values—which were inseparable from how participants understood and experienced their mental health problems, symptoms, suffering, and predicaments.

## Social support, stigma, and illness meaning

Although seeking and receiving support from first-degree family members and from close friends was identified by some participants, the stigma of mental illnesses was brought up frequently as a reason to avoid sharing their suffering with others in their social circles, particularly with extended-family members and friends. This problem was highlighted mostly by single women who were afraid of being labeled as “mad” and therefore unwanted in the context of their romantic relationships, marriage, and circle of friends, but it also was raised by men who were concerned about being labeled as weak and incompetent.

A participant who reported having the support of her family throughout her treatment, said:

Because of the respect I have for my parents [not wanting to upset them] I pretend that I have accepted my illness. But imagine that there is a girl who does not take these medications: she can meet someone and build a romantic relationship, but I cannot. I dated a guy, but I stopped it so as not to have to tell him that I am bipolar, to avoid hearing from him that I am crazy. … [I]n this society, even if a person is open-minded, when they get angry, they will tell me that “you are crazy.” I will break if I hear such a thing. … I decided to never date or get married to prevent such an experience happening, even though I would really like to become a mother (Participant F, a 27-year-old woman).

Participant G, a physician, described the potential impact of stigma on her career:

I never got the right reaction from people. In my workplace and other social circles, whenever I shared that I have a mood disorder and I take medication, I noticed people’s view of me changed and they took it as my weakness and judged me. For example, once, they said that she has problems and takes nerves medication [*ghors-e asâb*]. At first, sharing it was not important for me until I noticed that there are negative attitudes and they can affect my career. I decided to hide it from people. (Participant G, 32-year-old female, MD)

In general, we found that participants’ causal attributions and seeking support from their family members were related. Those who attributed their symptoms and suffering to their family environment, such as domestic violence, reported a lack of reliable family support and fear of being labeled and rejected by others in their social circle. Sometimes it was explicit how this social stigma and response exacerbated the problem. For example, Participant H stated:

For example, I am discussing something with my mom, not even fighting, and she immediately turns to me saying, “Go take your pill, you are not well, go to your bedroom.” This means, you are crazy, you are sick. As soon as I hear this, I become that bipolar, I explode. I know that not everyone who is angry is bipolar, but as soon as they tell me, “You are sick, you are bipolar,” I remember to become bipolar [she refers to being unpredictable and having outbursts.] (Participant H, 27-year-old female, BA)

Despite the dominance in Iranian culture of collectivist and familist values, the fear of receiving negative labels, of damaging personal and family social standing and reputation, and therefore becoming socially isolated may limit the safe space of family and social support that people need to rely on to cope with their mental health problems. Similarly, a study on mental health patients’ expectations for non-medical care in Tehran indicated that patients have an unmet need for programs that facilitate peer and social support and provide safe and confidential spaces for discussion by patients, caregivers, and professionals (Forouzan et al., 2011). Studies with caregivers have linked the difficulties that families have supporting their members who suffer from mental health problems to the wider lack of social support including emotional, financial, and professional resources, as well as the lack of information about mental health problems and interventions, which could empower families in providing care for their relatives with bipolar disorders (Shamsaei at al., 2010, 2015).

## Alternative pathways to care

Participants reported seeking alternatives to psychiatric medications and psychotherapy in order to both make sense of and cope with their mental health problems. One source of these alternatives has been greatly influenced by the Iranian diaspora. Compared to other diaspora groups, the Iranian diaspora is a more recent social phenomenon, a product of the large waves of emigration of mostly young and educated people whose lives have been directly and indirectly affected by the events of the 1979 Revolution, the Iran–Iraq War, and the 2009 Green Movement, among others (Ghorashi & Boersma, 2009). New technological advances and globalization have enabled the Iranian diaspora to build and maintain virtual ties with Iranians inside the country and across the world. Although this transnational connection is challenged by the Iranian state, diaspora–homeland relations influence the identity and lifestyle of people inside the country as well as the identity and integration of the immigrants in countries of migration (Cohen & Yefet, 2021).

In our study, participants in different cities and from different social backgrounds reported watching, listening to, and reading pop-psychology lectures given by Iranian psychologists in the diaspora (such as radio and TV shows by Dr. Holakouee^4^ and Dr. Farnoody^5^) as well as reading self-help lessons (mostly through social media and satellite TV programs) as common pathways to understand their psychological and interpersonal difficulties and obtain advice. In their live audio and video programs (both in recorded and online format), Iranian psychologists in the diaspora respond to people’s questions about their relational challenges, including marital and family problems, using psychological techniques drawing from systemic, cognitive-behavioral therapies, as well as acceptance and commitment therapy. Thousands of Iranians, both within and outside the country, reach out to these popular media figures to ask specific questions about their relational problems, receiving general answers that are, in some cases, more in line with the consultants’ values than those of the consultees or any empirical evidence. In some cases, the advice offered has no scientific basis and is socially contentious. For example, Dr. Holakouee’s advice against sexual relationships before marriage—calling this contradictory to “building love”^6^—has been criticized by many young people on social media as “old fashioned” and reproducing outmoded traditional norms.

While participants from more traditional cultural backgrounds reported visiting a prayer-writer, fortune-teller, or traditional healer, participating in *fara-darmani*^7^ (mystical philosophy, “psymentology,”^8^ metaphysics) workshops and using drugs (particularly marijuana) were alternative sources of help identified more often by younger participants in Tehran.

In our society, when people cannot do anything more [feel helpless], they turn to spirituality. I got onto a path that was called metaphysics courses [refers to *faradarmani*]. This stuff per se preoccupies you. You keep thinking about how to live differently from other people—e.g., if everyone eats with the right hand, I would practice eating with the left hand. Like swimming against the stream, which after sometimes [behaving differently] will break you [isolate you]. (Participant I, 28-year-old male, college degree)

Despite the extensive efforts from scientific (psychiatrists and psychologists), religious, and legal institutions to question, confront, and stop *faradarmani* approaches from becoming an alternative help-seeking pathway, many participants, particularly those interviewed in Tehran, heard about or personally tried joining the *erfan-e halgheh* (“Mysticism of the Ring”) as an alternative strategy. Although our participants reported that this strategy did not help them in the longer term, *faradarmani* and similar metaphysical approaches have become widely adopted in Iran as coping strategies used by mostly young and non-religious people.

The majority of participants in different cities mentioned religion and spirituality to be a strategy to cope with their emotional and psychological suffering; these strategies were identified as solitary praying, reading the Quran, and sending *salawat*^9^ (salutation, sending your blessing to Allah, Mohammad, and his family). Female participants gave varied accounts of why they were reluctant or refused to seek help from religious clerics, *akhoond-ha*. One participant attributed her lack of interest in seeking help from religious clerics for mental health problems to not trusting their intentions, having been sexually approached by several clerics, and not having the power to confront them without getting into trouble.

Although religion has an important place in people’s coping and help-seeking strategies, reflecting the diversity in Iranians’ lives, especially today when the conservative-liberal divide that runs through Iranian society is more significant than ever, religion is only one among many modes of explanation and help available to people when faced with adversity (Ahmadi et al., 2018).

## Barriers to and expectations from treatment

Among the perceived barriers to treatment, three sets of factors were identified, which can be categorized as *structural*, *biological*, and *stigma-related*. The most commonly reported structural barriers that influenced help-seeking and adherence included insufficient and expensive psychotherapy and counseling services, brief psychiatric visits focused only on symptoms and medication, and the lack of a therapeutic relationship due both to the brevity of visits and the lack of culturally informed practice. Health insurance in Iran does not cover mental counseling or psychological interventions. This might explain why psychiatrists keep their visits short, stay symptom-focused, and employ pharmacological treatment as the most common intervention (Dejman et al., 2009; Mianji & Kirmayer, 2020). This direct barrier to care for mental health problems has been reported to be a major factor in different countries (Esponda et al., 2020; Jain & Jadhav, 2009). Furthermore, despite the efforts of the state to control educational and clinical activities, particularly in the field of psychology, ensure they, the diversity in Iranian social and cultural norms does not necessarily follow the norms imposed by the state directly or indirectly through ideologically mandated educational and clinical practices.

The main barriers to treatment that related to biological factors, reported by the majority of our participants, were stopping therapy because of the side effects of psychiatric medications and fear of medication dependency. Encouragement from parents or other family to stop using medications was mentioned as another reason to stop treatment.

The stigma surrounding psychological and psychiatric disorders and the use of psychiatric medications was reported as a social factor that led to poor treatment adherence. In contrast, no participants reported concern about stigma for receiving counseling. Instead, most participants highlighted the need to have an affordable, safe, and non-judgmental clinical space to narrate their experience. Participants identified the need for community, social, and legal support to cope with their mental health problems, particularly those who attributed their BSD to family violence and social oppression.

## Discussion

This study identified five types of influence on the help-seeking strategies and trajectories of patients with BSD diagnoses in Iran: (i) structural limitations of the mental healthcare system; (ii) the modes of practice of biological psychiatry; (iii) the characteristics of the official psychology and counseling services permitted by Iran’s government; (iv) popular psychology and consultation (offered remotely) by Iranian psychologists and counsellors in the diaspora; and (v) alternative spiritual and cult-based groups.

The Iranian healthcare system promotes equal access to healthcare services and provides universal healthcare insurance for over 85% of the population; however, the coverage excludes non-medical interventions. Therefore, mental health services provided by the Ministry of Health, in both public and private sectors, emphasize the treatment of biomedical disease rather than mental health problems in order to benefit from insurance reimbursement policies (Fatemi, 2015). Only psychiatrists have prescription authority and can bill insurance companies for patient visits (Ghobari & Bolhari, 2001). This policy is an important contributor to disparities in the options people have in seeking help for their mental health problems. People with lower socioeconomic status and those who do not live in urban areas, where psychological and counseling services are expanding, often do not have access to psychosocial services, and the mental health interventions available to them are mostly limited to psychiatric medications prescribed by GPs or psychiatrists, which are covered by universal insurance (Fatemi, 2015).

In our study, although participants did not report any difficulties in terms of access to and affordability of seeing a psychiatrist, the majority expressed dissatisfaction with the services offered by psychiatrists because of the short visits, which focused mostly on pharmacological treatments, with little or no psychological intervention, leading to patients’ feeling unheard and misunderstood. This problem was reported mainly by patients who lived in rural areas or with lower socioeconomic status, who did not have access to psychologists and counselors in their regions and who could not afford to pay psychiatrists’ private fees for psychotherapy. In a previous article, we investigated psychiatrists’ explanations for offering only short visits and not providing psychological interventions (Mianji & Kirmayer, 2020). They acknowledged this mode of practice was not optimal but attributed it to economic factors such as a significantly lower fee for psychiatric visits compared to other specialties, the dominance of American biological psychiatry in Iran’s psychiatric training system, and the lack of collaborative mental healthcare services to refer patients to social services when severe problems such as domestic violence and abuse were revealed during the psychiatric visits (Mianji & Kirmayer 2020). We also described clinicians’ perspectives on help-seeking pathways of patients who received BSD diagnoses. Most of the psychiatrist participants described the tendency to seek help initially from GPs or neurologists as a barrier in treating their BSD patients (Mianji & Kirmayer, 2020). They claimed that neurologists and GPs often ordered unnecessary and expensive procedures like EEGs. Referring to GPs and neurologists before psychiatrists is associated with the stigma attached to seeking help from psychiatric services (Dezhman et al., 2008; Mianji & Kirmayer 2020).

Another important influence on patients’ help-seeking was the availability of counseling and psychological services offered by the official psychology and counseling sectors regulated by the Psychology and Counselling Organization of the Islamic Republic of Iran (PCOIRI). Seeking help from licensed psychologists and counsellors was reported mostly by participants who lived in large metropolitan cities—Tehran, Isfahan, and Shiraz—and by those with higher education and better economic status. Yet most participants reported low levels of satisfaction from the services provided and attributed that both to the high costs of the psychological service and the therapists’ lack of consideration of patients’ sociocultural and personal values. Although there has been an expansion of counseling and psychological services in Iran, these services are located mostly in urban areas and they remain limited or non-existent in rural areas (Fatemi, 2015; Sharifi, 2009). Furthermore, the focus of counseling and psychological services has gone through many changes in the past few decades due to the role that government-sponsored counseling and psychological organizations play as an agent and advocate of the regime’s sociopolitical agendas—rather than as a politically neutral, professional, and scientific component of the mental healthcare system.

Professional counseling in contemporary Iran started in the mid-1950s with a focus on improving the selection and evaluation of employees in different sectors, such as the Air Force, through the use of psychological measures developed in the Division of Psychological Research at the Ministry of Education. In the 1960s, the Iranian government sent a group of experts in education to Western countries to study counseling and psychology and transfer the knowledge to Iran. During the 1960s and 1970s, these experts, who had graduated from foreign universities, developed training programs in Iran’s universities in guidance and counseling, counseling psychology, family counseling, rehabilitation counseling, educational counseling, and career counseling as independent fields (Ahmadi, 2000). After the 1979 revolution, through what was called the “Cultural Revolution,” Iran’s Islamic regime closed all colleges and universities from 1980 to 1982, in order to Islamicize the education system. When the schools re-opened, an individual's adherence to the ideological and political principles of the Islamic regime became an important factor in admission to higher education and employment in different sectors, particularly the academic system (Sobhe, 1982). The psychological and counseling education and services were among the fields that were significantly affected by the political and ideological agenda of Iran’s regime in the 1980s. During this decade, counselors worked closely with religious Islamic clerics to provide mental healthcare services, particularly to military veterans who were serving in the Iran–Iraq War (1980–1988) (Fatemi, 2015). This influenced the type of interventions that counselors offered (Priester, 2008). Using religious concepts, such as trusting God and offering forgiveness, became a common language in psychological interventions when working with veterans who suffered from PTSD (Fatemi, 2015). In the 1990s, this approach was encouraged by PCOIRI, established in 1997, one of the governmental organizations mandated to follow the state’s political ideology and advocate for the integration of the religious and conservative cultural beliefs in psychological interventions. The importance of psychological language and practice as a vehicle for promoting the regime’s ideological and political values in society was acknowledged by the Iranian regime; hence, governmental organizations such as PCOIRI have explicitly emphasized adjusting counseling and psychological practices to conform to Islamic cultural values.^10^ Emphasizing the importance of family values and heterosexual marriage through offering pre-marital counseling became one of the ways to reach this goal (Fatemi et al., 2015).

In the new millennium, however, through widespread access to the Internet and satellite technology, Iranian society has been experiencing a transition from a society that is traditional, patriarchal, and religious to one that is adopting modern and secular values and that emphasizes individual rights (Mahdavi, 2007; Shahhosseini et al., 2012). This societal transition has led to a large cultural gap between generations and created interpersonal tensions at different levels, including parent–child relationships (Fatemi et al., 2015) and the relationship between social and legal authorities and ordinary people. Furthermore, these societal changes have polarized the attitudes of Iranian scholars in the field of psychology on the role of cultural adaptation in counseling and psychological practices. While some scholars and practitioners emphasize localizing psychological practices and tailoring the interventions to Islamic cultural values, such as the importance of family values and heterosexual marriage (Khalili, 2004), secular scholars have highlighted the lack in cultural adaptation in psychological interventions in order to advocate for and address the needs of individuals with less traditional and religious values, the needs of women in the context of gender disparities and inequality in social and legal rights, and the needs of LGBTQ individuals (Dezhamkhooy & Yazdi, 2013; Khoshnood et al., 2008; Najmabadi, 2011).

Participants in our study highlighted the lack of cultural sensitivity and secular approaches in psychological and counseling services as one of their main problems with the psychological services. The importance of taking into account the differences in patient and provider perspectives and the essential impact of these differences on help-seeking, treatment adherence, satisfaction, and outcomes has been emphasized in studies on explanatory models of illnesses (Kleinman & Benson, 2006; Tryon & Winograd, 2011). Understanding sociocultural and moral factors that are linked to patients’ perspectives on their suffering can guide the provision of culturally competent services, which may lead to greater treatment satisfaction (Kleinman et al., 1978). Attention to culture in mental health services is also a human rights issue insofar as individuals need to be able to realize their recovery, wellbeing, and aspirations in particular social and cultural contexts. However, the situation in Iran described by our participants illustrates that attention to cultural context in mental health services may also raise thorny human rights issues when individuals’ values come into conflict with the state’s political and social agenda centered on traditional and religious values.

Advances in technology in the new millennium—especially the Internet and satellite dishes—have connected Iranians inside the country to those in the diaspora to a far greater extent than before. Over 90 percent of Iranians have satellite dishes and access to Farsi-speaking TV channels that are based in other countries,^11^ mostly in the UK and the US, including BBC Persian, Iran International TV, Manoto, and Farsi VOA among many other media channels. The majority of Iranians use these sources to access information and for entertainment as alternatives to Iran’s national media, which is heavily censored and tightly controlled by the regime. This has influenced Iranians’ social and cultural values and expectations as well as the strategies people use to address everyday challenges and overcome problems. Indeed, consulting with and receiving general advice from popular Iranian psychologists in the diaspora via these media channels as well as their personal websites and social media pages has become one of the common strategies people use to address their mental health problems. Although mental health professionals encourage people not to view the pop-psychology channels as a replacement for psychiatric and psychological consultation, given the failure of psychosocial services to respond to the population’s mental health needs—whether due to structural incompetency or cultural insensitivity—the widespread use of pop-psychology lectures and interviews is not surprising. These media influence the populace as a whole by promoting a public conception of personal, interpersonal, and social possibilities and limitations, needs, expectations, and norms, which are substantially different from the values and norms of Iran’s ideological regime. In addition to Internet pop-psychology, *faradarmani*, a form of mysticism offered through series of seminars and workshops inside the country by Taheri (before his imprisonment) and by his followers (while he was in the prison),^12^ was reported by our participants as an alternative source of help for their mental health problems. The diaspora media channels and the promotion of *faradarmani* have been criticized and suppressed by the Iranian state, which views the diaspora media as “enemies of Iran’s revolution and regime” and *faradarmani* as apostasy and a threat to the Islamic ideology. The regime’s critique of *faradarmani* has been supported by Iranian mental health academics, who have challenged the claims made by the *faradarmani*’s leaders as lacking scientific evidence and have raised concerns about the danger that promoting non-scientific beliefs about mental health poses to psychiatric patients who need evidence-based interventions.

## Limitations

This study has several design features related to sampling and the interview process that may have influenced its findings. We interviewed patients who were seeing psychiatrists, who had received a diagnosis of BSD, who were selected by their clinician as potential participants, and who agreed to participate in the study and talk to the interviewer, an expat Iranian psychologist, at a time of political repression and turmoil. All these factors contribute to potential biases in the sample and in the kinds of narratives the patients provided. While for some participants, being interviewed by an expat psychologist who was not affiliated with Iranian universities and government might have made them feel more secure in sharing experiences that included criticism of the system, the fact that they were referred to the study by their current clinicians might have left them with concerns that their relationship with their psychiatrist would be affected by their comments in the research interviews, despite assurances of confidentiality.

## Conclusion

This qualitative study suggests that the help-seeking strategies of people diagnosed with BSD in Iran are influenced more by the structure of the healthcare system and the availability and affordability of existing options than by their own illness explanatory models. Although many Iranian patients attributed their mental distress to family and social determinants and hoped to receive psychosocial interventions, similar to other studies in Iran (Behrouzan, 2016; Ghanizadeh et al., 2008), patients reported that seeing a medical doctor and receiving biomedical intervention was a common help-seeking strategy. While the integration of psychiatric services into the public health system in Iran (Mohit, 2009) and the affordability of the psychiatric visits and pharmaceutical treatment have a strong influence on people’s motivation to seek biomedical treatment, this cannot be separated from other local sociopolitical factors—including the state’s efforts to integrate religious and ideological values into both the training and practice of mental healthcare providers—and global factors, including the impact of the larger international trends towards biomedical psychiatry and rapid circulation of information as a result of the Internet and social media (Smith, 2020). Similar to other countries with a totalitarian regime (Grigorenko et al., 1997), Iran’s government understands the importance and use of psychological language and practice as a means for spreading ideological and political values that are meant to facilitate social control. Serious challenges to both cultural and structural competencies in mental health services are posed by the government’s role in regulating and controlling the practice of counsellors and psychologists through both non-secular training and ideologically oriented interviews that are conducted as part of the licensing and employment of healthcare providers.^13^ The high costs and unequal distribution of psychosocial services in the country prevent many people from accessing other forms of care. These challenges affect the availability and quality of patient care and, especially, the respect for patient autonomy in the clinical negotiation (Forouzan et al., 2016). Given the lack of cultural and structural competence in the existing mental health services, patients turn to alternative strategies such as *faradarmani* and online pop-psychology content, which introduce other challenges, including the lack of scientific validity in the former and the risk of commercializing mental health needs and services in the latter.

In a globalized world, the help-seeking strategies and treatment expectations of patients with mental health problems, including BSD, cannot be understood without examining the intersection of global trends in mental health, individual predicaments, and the cultural and sociopolitical realities of local contexts. Current global mental health efforts that aim to promote care that is not confined to a narrow biomedical or psychiatric model must be designed to be participatory and person-centered, situating patients’ experiences in their cultural, social, economic, and ecological environments (Bemme & Kirmayer, 2020).

The strategies and barriers to accessing and utilizing proper mental health interventions examined in this study indicate that although incorporating psychosocial interventions into the public health system could be a positive step towards improving the accessibility and affordability of the mental healthcare services, to improve the quality of services it is essential to have a cultural and structural reform within the healthcare system that (1) prioritizes human rights and individuals’ values over the political and ideological values of the state and (2) promotes secular training of the mental healthcare providers in psychological and ecosocial models of care (Kirmayer, 2019).

Taken together with the findings reported in our previous papers (Mianji & Kirmayer, 2020, 2021), this study reveals how the globalization of biological psychiatry may inadvertently reinforce a lack of attention to culture and context in diverse settings including middle-income countries like Iran. There has been growing interest in expanding the biopsychosocial model to include structural determinants of health (Weiss et al., 2021). To realize this shift in perspective, we need strategies to make person-centered and ecological models of care applicable and feasible in non-Western countries, including theocratic and repressive states that do not recognize or prioritize human rights and are reluctant to confront structural inequities. Given the limited evidence for the cross-cultural applicability of global mental health approaches (Bemme & Kirmayer, 2020; Mills, 2022; Osborn et al., 2020), there is a need for continued research to study whether and how such models can be adapted and applied in different sociopolitical and cultural contexts in order to create greater health equity and social justice.

## Notes

1. Depakote (divalproex sodium) is a drug commonly used as a “mood stabilizer” for BSD.

2. In this interview, after clarifying the term “neurologist,” it was apparent that the patient meant “psychiatrist” but used “neurologist” in order to reduce the stigma.

3. Imam Hossein was the third Shia Imam, a grandson of the Islamic prophet Muhammad and an important religious figure in Islam.

4. Farhang Holakouee, known as Dr. Holakouee, is an Iranian licensed Marriage and Family therapist. He holds master’s degrees in Psychology and Economy and a PhD in Sociology. Holakouee lives in Los Angeles, California, and is the founder of the Beverly Hills Center for Well-being. Since March 6, 2000, Dr. Holakouee has been hosting “Mysteries and Needs,” a two-hour radio and TV program on Persian radio and TV satellite stations. His radio and TV programs and audio and video clips have become well-known and widely used by Iranians both inside and outside of Iran (https://en.wikipedia.org/wiki/Farhang_Holakouee).

5. “Nehzat Farnoody, Ph.D., is an Iranian Licensed Clinical Psychologist with a Specialty in Cross-Cultural Psychology, based in Los Angeles, California. From 1989 to 1992 Dr. Farnoody hosted the first Iranian-American Radio and TV program on psychological topics related to the needs of the Iranian-American community. From 1995 to 2001 she hosted several radio and TV psychology media literacy programs and, since 2002, has been the host of a popular satellite TV Talk Show called, “Inner Sight (Negahi be Daroon)” on American Farsi Network (www.AFNL.com) where she has an opportunity to reach out, educate, and raise awareness on Mental Health and psychological principles of parenting and healthy families for the Iranian-Americans, as well as the Iranians living in the global community” (https://drfarnoody.com).

6. https://www.youtube.com/watch?v=-EK8HntCWXw.

7. *Faradarmani* refers to a contemporary school of complementary and alternative medicine in Iran founded by Mohammad Ali Taheri, who attributed mental illness to a complex mechanism initiated by a person’s worldview, radiation (energy), and non-organic/supernatural beings (*Jinn*), also called “non-organic viruses” (Taheri & Yeghaneh, 2012), that enter and occupy the human body and sabotage their sanity (http://www.asreandisheh.com/Home/Content/103). Taheri was arrested in 2010 on charges of “acting against national security” and has been in prison since 2011. He was sentenced to death in 2015 by the revolutionary court on charges of Fesad Fel Arz (corruption on earth) and establishing a “diversionary cult” (https://www.nytimes.com/2017/08/28/world/middleeast/mohammad-ali-taheri-iran-death-sentence-mystic-healer-shiite-mystic.html). He was eventually released in 2019 and granted asylum in Canada in 2020, where he now resides. After his arrest, the Iranian government established *Nejat Az Halghe* (“The Ring Rescue Organisation”) to help “survivors” of Taheri’s teachings called *erfan-e halgheh* (Mysticism of the Ring). “Abbas Ali Allahyari, head of Psychology and Counselling Organisation of the Islamic Republic of Iran (PCOIRI) and associate professor of Tarbiat Modares University has described the teachings as dangerous, predatory and exploitative, especially towards mentally ill people. Taheri’s practice involves the exorcism of jinn. Dr. Nader Mahluji, a prominent psychiatrist in Tehran stated that he believed in jinn, as they are described in the Quran, but that he does not believe they interact with humans because this is not mentioned in the Quran, going on to state, ‘People who claimed to have been possessed by jinn were either charlatans, delusional, or had undergone some form of dissociation. Some may be schizophrenics and require medical treatment’” (https://en.wikipedia.org/wiki/Mohammad_Ali_Taheri).

8. According to Taheri and Bayyazi (2013), “Psymentology” (a neologism he coined from ‘psyche’ + ‘mental’ + logy] or “Interuniversal Mind-Psychology” refers to an “Interuniversalist perspective on the human being with an all-inclusive approach. In Psymentology, mind and psyche are each addressed as a separate concept, comprising a specific part of an individual. The main target of Psymentology is understanding and gaining knowledge about human being, the constituent software of human existence, diagnosis and treatment of psymental(mental and psychological) disorders, as well as other related unidentified problems. Psymentology is based on the theory of “Consciousness Bond of the Parts” or “Parts Having Consciousness in Common.” “Through this therapy, patient becomes connected to Interuniversal Consciousness via psymento-therapist. After this bond is established, he/she automatically undergoes Scanning stage in which all existential constituents of individual undergo scrutiny through Consciousness Bond in order to detect any manifested or hidden diseases” (p. 1534).

9. There is a tradition of repeating *salawat* from five to thousands of times, which for many people has a grounding function, like repeating a mantra.

10. “To increase heterosexual marriage success rates, the Iranian government mandated that couples who are getting married receive 16 hours of counseling prior to their heterosexual marriage. In premarital counseling, couples participate in genetic, health, and marriage counseling (Salarvand, Bahri, Heidary, & Khadive, 2011), which can include different counseling opportunities, such as face-to-face counseling and watching psychoeducational videos with other couples” (Fatemi, 2015).

11. https://www.isna.ir/news/94031307607/بیش-از-90-درصد-ایرانیان-ماهواره-دارند-شکسته-شدن-قبح-ها-با-پخش

12. Mohammad Ali Taheri, the leader of *faradarmani* (see note 9), resumed his classes and seminars through satellite, internet, and social media channels after his release from prison and resettlement to Canada.

13. One of the prerequisites for obtaining licensure for clinical practice and for being employed in any governmental and official sector is an interview to evaluate the person’s loyalty to the regime’s ideological and political objectives and values, conducted by the non-scientific committees in all official and governmental organizations in Iran including PCOIRI. Although what clinicians state during the interview may not reflect all of their personal and political values, verbally committing to submit to the state’s values as a way to protect their licensure and career prospects may limit their ability to adjust their practice to the personal values of the patient, even without ongoing scrutiny of their practice.
